# The Limits and Influences of a Nurse's Vocation

**Published:** 1888-01-07

**Authors:** R. Brudenell Carter

**Affiliations:** Ophthalmic Surgeon, St. George's Hospital, and the National Hospital for Paralysed and Epileptic, etc.


### The Limits and Influences of a Nurse's Vocation.

A Lecture by R. Brudenell Carter, F.R.C.S.,

Ophthalmic Surgeon, St. George's Hospital, and the National Hospital for Paralysed and Epileptic, etc.

I have been asked to come here this evening in order to address to you some remarks introductory to a course of lectures on the special duties of nurses, which has been promised to you by the medical officers of your institution. I have obeyed this call with peculiar pleasure, because I think it would be difficult to over-estimate the value of good nursing to the community, and because I think it must be a help to nurses, as it is to the followers of any other calling, to be assured, from time to time, that those who have the best opportunities of seeing their work are fully alive to its usefulness and to its importance. It has lately been my business to write a report on the progress made by medicine and surgery during the reign of Her Majesty the Queen, and in that report I have had to bring into prominence the fact that the rate of mortality among the English population has, in the 50 years, been diminished in a very remarkable degree. To take a few examples, I may tell you that, in the five years from 1838 to 1842, or in the first five years of the Queen's reign, London had an average population of 1,840,865 persons, and an average annual mortality of 2,557 in every hundred thousand. In the five years from 1880 to 1884, the last of the ordinary five-year periods for which the returns have been completed, London had an average population of 3,894,261, and an average annual mortality of 2,101 in every hundred thousand. If the rate of mortality in the second of these periods had been the same as in the first, 17,328 people would have died in London in each of the last-named five years, who, as events actually occurred, were preserved alive. The mean annual death-rate for the whole of England and Wales from 1838 to 1847 was 22·16 per thousand; and the mean annual death-rate from 1876 to 1885 was 20·07 per thousand. If the mortality in the latter period had been as high as in the former, the deaths would have been more numerous than they were by over half-a-million, or, to speak precisely, by 550,191. In the year 1885, taken alone, the death-rate for England and Wales was 19·0 per thousand, which, on an estimated population of twenty-seven millions, implies that the deaths during the year were less numerous by 48,900 than the average annual number during the ten years which the year in question brought to a close.

In the report from which I quote these figures I have also had to say that the vast improvement which they indicate must be due partly to preventive medicine and partly to curative medicine, but that it is impossible exactly to divide the credit between these two great branches of the healing art. We know that improved sanitary measures and greater cleanliness have done much to prevent disease, and we know that improved methods of treatment have done much to cure it, but we cannot say how much of the total improvement has been due to one of these influences, and how much to the other. Neither, in endeavouring to estimate the amount of life-saving which has been due to improved methods of treatment, can we say how much should be attributed to the better guardianship of the sick-room, and to the better and more complete carrying out of all medical instructions, for which we are indebted to trained nurses. That I have not been unmindful of the degree in which they are entitled to participate in the general credit may be shown by another quotation from the report in question, in which I have used these words:—

"The application of improved medical and surgical knowledge in the sick-room has been greatly facilitated by the education and training of professional nurses; a work which was commenced by Miss Nightingale as one consequence of the lessons taught by the Crimean War. These nurses now form a numerous and invaluable body of public servants, discharging their important and arduous duties with an amount of care and self-devotion of which it would be difficult to speak too highly."

Before it is possible to speak profitably upon any subject, it is necessary to obtain some idea of its nature and boundaries, of what it includes and of what it does not include; and I must confess that I have found it difficult to frame any satisfactory definition of nursing. It borders, on the one hand, upon the care which people who are in the possession of good health ought to take in order to preserve it; and it borders, on the other hand, upon preceedings which many would claim as being distinctly medical or surgical. But I think we shall arrive at some conception of the truth if we remember that the treatment of all disease has for its object the removal of some condition which is oppressing the patient, and is making him or keeping him ill. A patient has inhaled or swallowed, or in some way received into his system, some poisonous thing—it may be, for example, the poison of typhoid fever or of cholera; and the business of the physician is to find out the presence and the nature of this poisonous thing, and then to endeavour to diminish its effects while it is in the body, and to provide for its expulsion by natural channels as soon as may be. If these objects can be satisfactorily attained, when the poison is got rid of the patient will be likely to get well. In like manner, a patient may be suffering from the presence of some diseased part, say from dead bone in his leg, which keeps him awake by pain and exhausts him by discharge. It is the business of the surgeon to find out the presence of this dead bone, and in due time to take it away, and when it is taken away the patient may recover. In these cases, as in all others, it is incorrect to say that the doctor cures the patient. The doctor simply does something which allows the patient to get well, places him in favourable conditions for recovery, and Nature does the rest; or, if Nature fails to do it, the patient dies in spite of the doctor. In the two cases supposed, there would be in each some particular thing which was bad for the patient, and which prevented or retarded his recovery,—the poison in the medical case andthe dead bone in the surgical case—and the removal of these things was the province of medicine or of surgery. But to lie in a stuffy and badly ventilated room, or among dirty surroundings, would have been equally bad for either patient; and I think I should describe the province of nursing as being the removal of conditions or influences which are bad for all sick people, which would retard or prevent the recovery of all; while the province of medicine or surgery is the removal of conditions or influences which exist only in particular cases. If you will accept this as an account of the proper limits of your vocation, we have next to see what the influences are which may be described as universally or generally injurious to the sick. They may be summed up, I think, as neglect of cleanliness, neglect of ventilation, neglect of regular feeding, neglect of temperature, neglect of quietness, neglect of position, neglect of comfort, and neglect of watchfulness. Let us take them briefly in their order.

Neglect of cleanliness—or, to put the matter more simply, the presence of dirt—is among the most fatal and the most far-reaching of all the influences which prevent or retard the recovery of patients. It may obviously exist in two different ways, either as want of cleanliness of the person of the patient, or as want of cleanliness of his surroundings; but the two forms fade imperceptibly into each other, and, where one is present, the other is at least not far away. In order to understand the noxious effect of dirt it is necessary to know that all separated portions of an animal body, as soon as they no longer form parts of a living structure, begin to enter upon a state of decomposition, and that in a certain stage of decomposition they are all poisonous, some of them very actively so. Sometimes they may be the carriers of some particular form of poisoning, which we call infection; as when fragments of peeling skin are the carriers of the poison of scarlet fever, or the matters which pass away from the bowels are the carriers of the poison of typhoid fever. But, apart from all special influences of this kind, a piece of dead animal matter, which is necessarily beginning to enter upon a state of decomposition, has the property of hastening the commencement of decomposition in the structures of a still living body with which it may be brought into contact; and it is in this way that animal dirt is liable to induce putrescent changes in wounds into which it is accidentally or carelessly introduced. Within my recollection, there was a ward in a London hospital in which the results of surgical operations were singularly unfortunate; and it came at length to be observed that there was one bed in this ward the occupant of which never recovered. To be placed in this bed was a sentence of death; and the peculiarity of the bed was at last found to be that it stood close to a window underneath which, on land not belonging to the hospital, there was a dustyard, a collection of the street sweepings of London—a collection containing, as a matter of course, a vast amount of decaying animal matter. When the window was open, or even, for the matter of that, when it was not open, portions of decaying animal matter would find their way into the ward, and would alight, in the greatest quantity and with the greatest frequency, upon the bed and the patient nearest to their point of entrance. Such portions of decaying matter, finding access to operation wounds or others, converted them from a healing into a putrescent state, occasioned the conditions called "hospital gangrene" and so forth, and caused the patients to poison themselves by the absorption into their blood of their own putrescent wound discharges. The conditions which were present in an intense degree in this ward, and over this particular bed, are present, in a lesser degree, in every bed-room in London, or wherever there is a crowded population.

*(To be continued.)*

